# Status of mandatory treatment of mentally ill offenders without criminal responsibility in China: Information from 5,262 mandatory treatment judgments

**DOI:** 10.3389/fpsyt.2023.1129954

**Published:** 2023-04-03

**Authors:** Yu-Feng Qiu, Xiao-Tong Yin, Zi-Ye Wang, Rui Yang, Jeremy Coid, Xia-Can Chen, Jun-Mei Hu

**Affiliations:** ^1^West China School of Basic Medical Sciences and Forensic Medicine, Sichuan University, Chengdu, China; ^2^Brain Research Center and Mental Health Center, West China Hospital of Sichuan University, Chengdu, China; ^3^Wolfson Institute of Preventive Medicine, Queen Mary University of London, London, United Kingdom

**Keywords:** criminal responsibility, mandatory treatment, the judgment documents, the criminal procedure law, patients with mental disorder, COVID-19 pandemic

## Abstract

**Background:**

To avoid public health risks, all governments ensure monitoring and treatment of mentally ill persons if they offend and assess their level of criminal responsibility. The Criminal Procedure Law of the People’s Republic of China (2013) instituted special procedures. However, there are few articles in English which explain the implementation of mandatory treatment procedures in China.

**Methods:**

We collected 5,262 qualified documents from 2013 to 2021 from the China Judgments Documents Online. We analyzed social demographic characteristics, trial-related information as well as the mandatory treatment-related content, to investigate the mandatory treatment of China’s mentally ill offenders without criminal responsibility, from 2013 to 2021. Simple descriptive statistics and chi-square tests were used to compare differences among several types of documents.

**Results:**

There was an overall change trend of the number of documents: increasing year by year from 2013 to 2019 after the implementation of the new law, but with sharp decrease in 2020 and 2021 during covid-19 pandemic. From 2013 to 2021, a total of 3,854 people had applications made for mandatory treatment, of whom 3,747 (97.2%) were given mandatory treatment, 107 (2.8%) had applications rejected. “Schizophrenia and other psychotic disorders” was the most common diagnosis in both groups and all offenders receiving mandatory treatment (3,747, 100.0%) were considered to have no criminal responsibility. A total of 1,294 patients had applications made for relief of mandatory treatment, of whom 827 (63.9%) were subsequently approved for relief, 467 (36.1%) were rejected. A total of 118 patients had applications for relief two or more times, and 56 (47.5%) were finally relieved.

**Conclusion:**

Our study presents the Chinese model of a criminal mandatory treatment system to the international community which has been in operation since the implementation of the new law. Legislatory changes and covid-19 pandemic can have effect on the number of mandatory treatment cases. Patients, their close relatives and mandatory treatment institutions have the right to apply for relief from mandatory treatment, but the final decision in China is taken by the court.

## 1. Introduction

World Health statistics 2022 showed that almost one billion people globally suffer from at least one mental disorder (1). The China Mental Health Survey (CMHS) found that the weighted lifetime prevalence of any mental disorder, excluding dementia, in China is 16.6% (2). A subgroup of patients with mental disorder are violent, with harmful consequences to society (3). One study found that in terms of general criminality, there are few differences between patients with mental disorder and the average population; it is only with regard to violence that the risk clearly increases, and the effect is particularly pronounced in the case of homicide (4).The potential dangerousness of these patients’ violent behaviors should not be under-estimated. Patients with mental disorder who have been legally identified as without criminal responsibility need to receive guardianship and treatment (5).

However, proper and dignified treatment of offenders with mental disorder requires the combined efforts of the criminal justice system and the mental health system, guided by continuous improvement of relevant laws and regulations. Different countries have different legally prescribed norms for psychiatric evaluation and disposition of mentally ill offenders. However, there are many similarities in the relevant legal provisions which set out the principles of mitigating sentences for such offenders (6). Based on the results of a forensic psychiatric assessment, a defendant without criminal responsibility can be placed in hospital for treatment rather than receiving a custodial sentence in most countries with adequate mental health provision. Similar to the other countries, forensic psychiatrist in China also assess the defendant’s mental state and whether there is any kind of mental illness and give opinions on criminal responsibility. The court will also formally refer cases for forensic psychiatrist opinions to determine whether the defendant is full, partial, or having no responsibility for the offence due to mental illness.

In China, there is a long history of legislation which reduces severity of punishment of mentally ill offenders whilst requiring family members to care for the individual. This can be traced back to Western Zhou Dynasty (about 2,000 B.C.) (7). By the Qing Dynasty (1644 to 1912), the law clearly stipulated that the family, neighbors, and patriarchs of a patient with mental disorder had a duty of guardianship over him/her. If the patient with mental disorder subsequently killed someone, his/her guardian would be punished for failing to supervise him/her (7).The traditional Chinese concept of family was that family members were closely related to each other and therefore have responsibility for the behavior of the family. The legal system of guardianship was also used to place the responsibility for the care on the guardian. Guardians were later required to take care of and watch over patients with mental disorder, and actively cooperate in seeking medical treatment according to article 15 of the Criminal Law of the People’s Republic of China (PRC), promulgated in 1979 (8, 9). This article emphasized the responsibility of the guardian within the legal aspect, by stipulating that: *a patient with mental disorder who causes dangerous consequences when he/she is unable to recognize or control his/her own behavior is not criminally responsible; however, his/her family members or guardians should be ordered to keep him/her under strict surveillance and arrange for his/her medical treatment.* However, because guardians lacked knowledge of mental illness and did not always have the ability to supervise and restrain patients with mental disorder (10), such patients could endanger public safety again in the future. New legal provisions were subsequently introduced.

**Figure 1:**
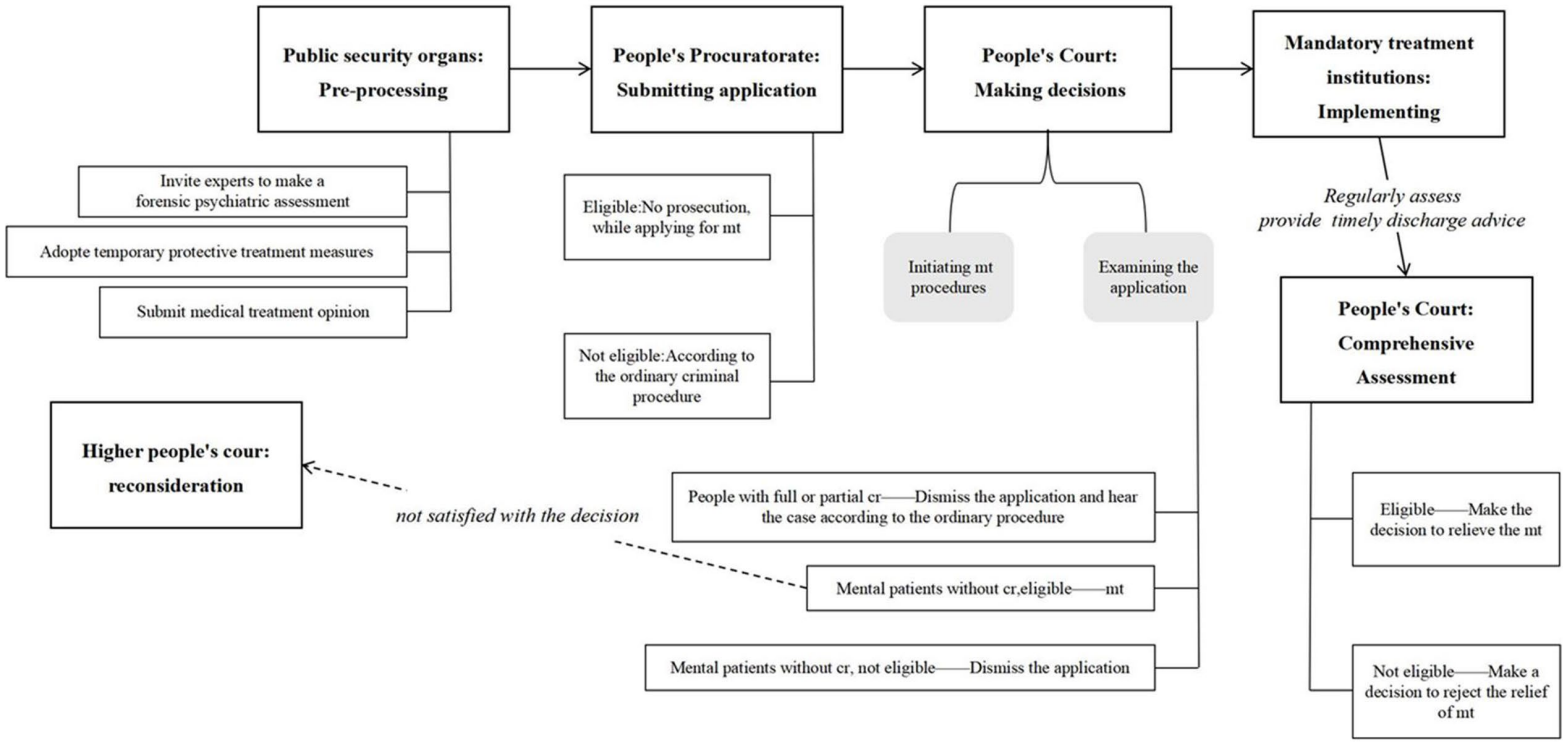
Logical structure of mandatory treatment procedures. mt, mandatory treatment; cr, criminal responsibility.

The Criminal Law in 1997 further amended and supplemented the treatment of mentally ill offenders by proposing that “If a patient with mental disorder causes harmful consequences at a time when he/she is unable to recognize or control his own conduct, upon verification and confirmation through legal procedure, he/she shall not bear criminal responsibility, but his/her family members or guardian shall be ordered to keep him/her under strict surveillance and arrange for his/her medical treatment. When necessary, the government may compel him to receive medical treatment.” The notion of “mandatory treatment” was introduced here for the first time and the mandatory treatment system officially entered the criminal law field in China. However, no specific procedural norms were proposed and the condition of “*when necessary*” was not clearly explained. The criteria for application of mandatory treatment were ambiguous, and the outcome of the court’s disposition of mentally ill offenders without criminal responsibility was susceptible to public questioning (11).The Criminal Procedure Law of China for the second version was formally implemented on January 1, 2013. This filled a gap in the procedural law of China’s criminal mandatory treatment system by setting up a special procedure for “*the mandatory treatment of mentally ill offenders who bear no criminal responsibility according to the law*”(Figure 1). Article 284 of the Criminal Procedure Law (2013) provides that “*a patient with mental disorder who commits violent acts, endangering public security or seriously harming the personal safety of citizens, and who is identified by legal procedures as not criminally responsible according to law, may be subject to mandatory treatment if there is a possibility of continuing to endanger society*.” This article specified the object and conditions of application of mandatory treatment. Subsequently, the Public Security Bureau, the Procuratorate and the Court (collectively referred to as the case handling organs) issued a series of provisions to further detail the implementation of the procedure.

When it is suspected that the perpetrator is not guilty by reason of mental disorder and needs mandatory treatment, the public security agencies can submit a mandatory treatment opinion to the people’s procuratorate. The mandatory treatment opinion is a type of legal instrument. It sets the basic facts of the case, the identification of the crime, the treatment of opinions and reasons, as well as the legal provisions based on. After evaluation, the people’s procuratorate apply to the people’s court for mandatory treatment (5). If the public security agencies transferred or prosecution found that the mentally ill offenders are eligible for mandatory treatment in the process of evaluation, the people’s procuratorate can also directly apply to the people’s court for mandatory treatment after forensic psychiatric assessment. The court then needs to conduct a comprehensive review of the violent or illegal behavior of the perpetrator, the original forensic psychiatric assessment opinion, the level of dangerousness to society, and the supervisory ability of the guardian before making a decision on mandatory treatment (12). For mentally ill offenders who do not meet the conditions of mandatory treatment but have no criminal responsibility (low societal risk, or where guardians and close relatives have the ability to look after the individual, etc.), the court can make a decision to reject the application for mandatory treatment, but order their close relatives or guardians to closely supervise and provide medical treatment. For respondents with full or part-diminished (but not lacking) criminal responsibility, the court would make a decision to reject the application for mandatory treatment and return it to the people’s procuratorate for processing according to law.

In China, for mentally ill offenders without criminal responsibility, the government arranges for their mandatory treatment in forensic psychiatric hospitals (Ankang Hospital). These are similar to medium-high security hospitals in the UK and maximum security hospitals in North America (5, 13). Practice shows mandatory treatment is carried out in the form of hospitalization throughout the country (14–16). Swiss criminal law divides mandatory treatment into two types: outpatient treatment and hospitalization. According to Articles 59 and 63 of the Swiss Penal Code, outpatient treatment is applied as a priority, and hospitalization is applied only when necessary (17). In France, patients without criminal responsibility by decision of the court are also required to undergo mandatory outpatient treatment at the end of involuntary hospitalization (18).

The duration of mandatory treatment is clearly defined in many countries, such as Germany, Switzerland and the United States (19–21). In China, the duration of mandatory treatment is not certain. It requires the staff of the forensic psychiatric hospitals to regularly evaluate the disease status and personal dangerousness of the mentally ill offender. When the evaluation reveals that the mentally ill offender ‘s condition is in remission and there is no risk of harm, hospitals should promptly submit opinions to the people’s court on the application for relief of mandatory treatment. The offender subjected to mandatory treatment or his/her close relatives also have the right to apply for the relief of mandatory treatment if they believe that they should not be subject to mandatory treatment, or that they are no longer a danger to the public following treatment (22). The court will assess whether he/she is “physically dangerous” in terms of his/her diagnostic assessment report, the forensic psychiatric assessment opinion, and his/her close relative’s current guardianship ability, and make the decision to relieve or continue his/her mandatory treatment.

**Figure 2:**
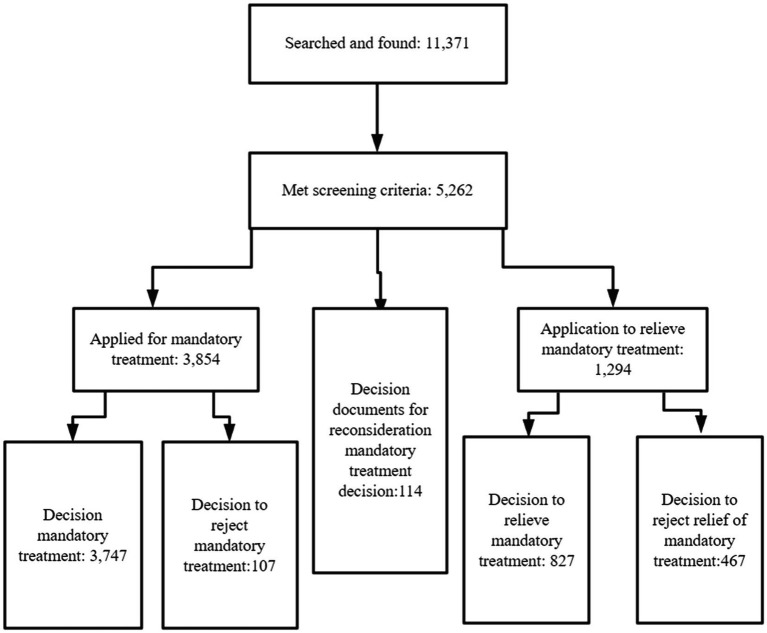
Flow chart of document classification.

The aim of this study is to describe mandatory treatment procedures in China following a finding of diminished responsibility for criminal behavior in court. We collected relevant judgment documents on China’s Judicial Documents Online for a 9 year period from 2013 to 2021, and divided them into five types of documents according to different procedural stages (see Figure 2). We extracted socio-demographic, court trial, forensic psychiatric assessment, and mandatory treatment care-related information from the documents, analyzing the differences between those who were subject to mandatory treatment and those who were rejected for mandatory treatment. We then compared those who were relieved from mandatory treatment and those who were rejected for relief of mandatory treatment.

## 2. Methods

### 2.1. Materials

Data were collected from the judicial documents of the China Judgments Documents Online.^1^ In 2013, the Supreme People’s Court established a network of Chinese judicial documents which publishes effective judgment documents from the people’s courts at all levels. It covers different types of cases such as criminal, civil, administrative, as well as different trial procedures such as second instance, retrial and application for retrial. These cases are from all provinces and cities across China.

### 2.2. Procedure

The Criminal Procedure Law of the PRC (2013) was officially implemented on January 1st, 2013 (23). In order to reflect the enforcement of PRC on mandatory treatment, we collected the judgment documents from 2013 to 2021, by searching key words of “criminal cases” and “mandatory treatment” at the same time. Documents were downloaded by year, and we initially obtained 11,371 documents. The criterion was that the conclusion in documents must refer to mandatory treatment. We reviewed all documents, and ultimately only 5,262 documents met the screening criteria. The documents were divided into five types: mandatory treatment decision documents, documents of rejecting the mandatory treatment decision, documents of mandatory treatment reconsideration decision, documents of mandatory treatment relieving decision, and documents of rejecting the relief of mandatory treatment decision.

Using Epidata V4.6 software to input the basic information on mentally ill offenders in the document, including socio-demographic characteristics (name, gender, age at the time of crime, marital status, education level, nationality, whether they had employment); the type of crime involved [mainly including crimes of violating citizens’ personal rights and endangering social and public security (24)]; forensic psychiatric assessment information (Forensic psychiatric assessment institution, diagnosis, and the degree of criminal responsibility), in which the classification of diagnosis is based on the third edition of classification and diagnostic criteria of psychosis in China (CCMD-3, 5); mandatory treatment information (duration of mandatory treatment, place of mandatory treatment, whether to grant/reject mandatory treatment this time, applicant, reconsideration reasons and results, etc.). If the criminal suspect in a document suffers from multiple mental disorders or is suspected of multiple crimes only the diagnosis or crime which ranked first in the document were recorded. The missing items in the document are recorded as “not mentioned.”

### 2.3. Analysis

Statistical description was made for the basic information in all documents, the documents of the same person applying for relief of mandatory treatment multiple times, and duration of mandatory treatment. Chi-square tests were used to compare categorical variables between patients receiving mandatory treatment and those who were rejected, offenders who were relieved from mandatory treatment, and those who were rejected for relief of mandatory treatment. An alpha level of below 0.05 was defined as statistical significance. The missing values and missing rates of all items are listed in the supplementary table. All analyses were performed using Statistics Analysis System V9.4 (SAS9.4).

## 3. Results

### 3.1. Quantity information of various types of documents

**Figure 3:**
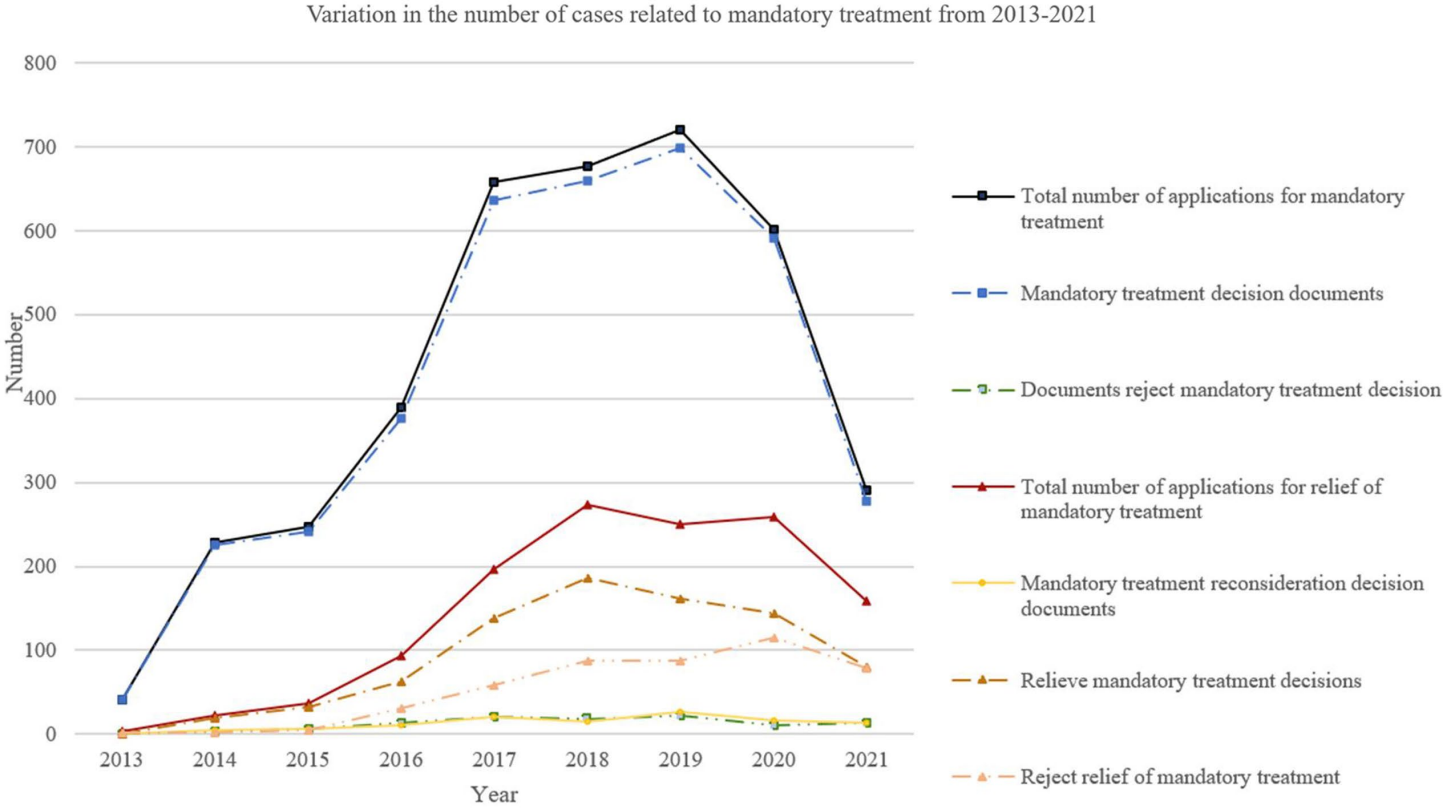
Variation in the number of mandatory treatment-related documents from 2013 and 2021.

From 2013 to 2021, 5,262 qualified documents were included in analysis (Figure 2). A total of 3,854 people applied for mandatory treatment, of whom 3,747 (97.2%) were approved for mandatory treatment, and 107 were rejected for mandatory treatment. A total of 1,294 mentally ill offenders applied for relief of mandatory treatment, of whom 827 (63.9%) were approved for relief from mandatory treatment, and 467 were rejected for mandatory treatment relief. The change of total number of documents over different years is shown in Figure 3. It can be seen from Figure 3 that the overall change trend of the number of documents was consistent, increasing year by year from 2013 to 2019, but with sharp decrease in 2020 and 2021.

### 3.2. Basic information on applying for mandatory treatment

All requests for mandatory treatment were submitted to the court by the Public Prosecutor’s Office. Over 9 years, a total of 3,854 offenders with mental illness had applications for mandatory treatment, of whom 3,747 (97.2%) received mandatory treatment, and 107 (2.8%) applications were rejected. Table 1 presents the comparison of basic information for the two groups. The distribution of diagnosis, the type of crime involved and criminal responsibility significantly differed between the two types of offenders. The forensic psychiatric assessment opinions on offenders under mandatory treatment were all “no criminal responsibility.” Offenders with partial criminal responsibility accounted for 11.2% of those who were rejected for mandatory treatment. Among the reasons for rejecting mandatory treatment, 45 (42.1%) mentally ill offenders were sent to the hospital for temporary restraint after committed an illegal act, and their condition had improved. The existing evidence in 13 (12.1%) documents was insufficient to prove that the suspect had committed any illegal acts, so they were acquitted.

**Table 1:** Descriptive characteristics of persons for whom mandatory applications were made (*N* = 3,854).

		Applied for mandatory medical treatment (total)		Received mandatory treatment		Rejection of mandatory treatment		χ ^2^
		*N*	%	*N*	%	*N*	%	
Total		3,854		3,747		107		
Sex								
	Female	696	18.1%	672	17.9%	24	22.4%	1.447
	Male	2,907	75.4%	2,831	75.6%	76	71.0%	
Age								
	<18	14	0.4%	14	0.4%	0	0.0%	3.932
	18–45	2,226	57.8%	2,158	57.6%	68	63.5%	
	46–65	915	23.7%	898	24.0%	17	15.9%	
	>65	111	2.9%	108	2.9%	3	2.8%	
Nationality								
	Ethnic Han	2,962	76.9%	2,878	76.8%	84	78.5%	0.017
	Others	296	7.7%	288	7.7%	8	7.48%	
Educational level								
	Illiterate/primary education	1,238	32.1%	1,201	32.1%	37	34.6%	2.763
	Secondary education	1,670	43.3%	1,627	43.4%	43	40.2%	
	Bachelor’s or higher level	194	5.0%	185	4.9%	9	8.4%	
Employment								
	In work	1978	51.3%	1922	51.3%	56	52.3%	1.776
	Unemployed	950	24.6%	931	24.8%	19	17.8%	
	Marital Status								
	Single (unmarried, divorced)	269	7.0%	259	6.9%	10	9.4%	0.202
	Married	1,103	28.6%	1,068	28.5%	35	32.7%	
CCMD-3 diagnosis								
	Organic mental disorders	190	4.9%	178	4.8%	12	11.2%	16.282*
	Mental disorders due to substances	141	3.7%	136	3.6%	5	4.7%	
	Schizophrenia and other psychotic disorders	2,902	75.3%	2,829	75.5%	73	68.2%	
	Mood (affective) disorders	170	4.4%	164	4.4%	6	5.6%	
	Hysteria, Stress-related disorders, Neurosis	17	0.4%	16	0.4%	1	0.9%	
	Personality disorders, Habit and impulse disorders, Psychosexual disorders	4	0.1%	4	0.1%	0	0.0%	
	Mental retardation, and disorders of psychological development	92	2.4%	86	2.3%	6	5.6%	
	Other mental disorders and psychological health conditions	103	2.7%	102	2.7%	1	0.9%	
The type of crime involved								
	Intentional homicide	1805	46.8%	1774	47.3%	31	29.0%	76.885 ***
	Intentional Assault	1,486	38.6%	1,449	38.7%	37	34.6%	
	Arson	251	6.5%	236	6.3%	15	14.0%	
	Robbery	70	1.8%	69	1.8%	1	0.9%	
	Endangering public safety	63	1.6%	61	1.6%	2	1.9%	
	Disrupting public service	57	1.5%	53	1.4%	4	3.7%	
	Intentional destruction of property	49	1.3%	40	1.1%	9	8.4%	
	Others	66	1.7%	60	1.5%	6	5.6%	
Criminal responsibility								
	No responsibility	3,842	99.7%	3,747	100.0%	95	88.8%	386.180***
	Partial responsibility	12	0.3%	0	0.0%	12	11.2%	

*p < 0.05, **p < 0.01, ***p < 0.001. Missing values and missing rates are shown in the supplementary table.

### 3.3. Basic information of the decision of mandatory treatment reconsideration

Between 2013 and 2021, there were 114 mandatory treatment reconsideration documents submitted to higher courts for refusing mandatory treatment. Among them, 113 reconsideration cases were tried by the intermediate people’s court and 1 case by the high people’s court. The reasons for applying for reconsideration were different: 40 (35.1%) applying on grounds of no longer being dangerous; 31 (27.2%) applying for the second assessment because of disagreement between parties or close relatives about the first forensic psychiatric assessment; 22 (19.3%) applying for guardians in the belief that they had sufficient capacity and could provide a suitable environment to effectively supervise the offenders, so that mandatory treatment was not necessary; 16 (14.0%) applying because parties or close relatives proposed that the facts identified by the court at the first instance were unclear and the evidence was insufficient. There were 108 (94.7%) documents rejecting the reconsideration of the application, 4 (3.5%) documenting the decision of the superior court to revoke the mandatory treatment and sending it back to the basic level court for retrial, and 2 (1.8%) applicants deciding to withdraw their applications.

### 3.4. Basic information of applying for relieving of mandatory treatment

From 2013 to 2021, there were 1,294 documents applying for relief of mandatory treatment, including 827 offenders (63.9%) who were relieved from mandatory treatment, and 467 (36.1%) who were rejected for relief. Table 2 lists the comparison results of the basic information for the two groups. The distribution of gender, marital status, diagnosis and the type of crime involved significantly differed between the two groups. Among the 827 patients relieved from mandatory treatment, 584 (70.6%) had applications from their close relatives, and 2 (0.02%) from the director of the committee where those receiving mandatory treatment were located. Among the 467 people who were rejected for relief of mandatory treatment, 321 (68.7%) had applications from their close relatives (The minimum duration of mandatory treatment is 1 month, and the maximum 96 months.)

**Table 2:** Descriptive characteristics of mentally ill offenders for whom relief of mandatory treatment applications were made (*N* = 1,294).

		Applied for relief of mandatory treatment(total)		Mandatory treatment relieved		Rejected for relief of mandatory treatment		χ ^2^
		*N*	%	*N*	%	*N*	%	
Total		1,294		827		467		
Sex								
	Female	258	19.9%	178	21.5%	80	17.1%	4.424*
	Male	971	75.0%	601	72.7%	370	79.2%	
Age								
	<18	7	0.5%	5	0.6%	2	0.4%	4.512
	18–45	867	67.1%	539	65.1%	328	70.2%	
	46–65	207	16.0%	139	16.9%	68	14.6%	
	>65	34	2.7%	26	3.1%	8	1.7%	
Nationality								
	Ethnic Han	1,014	78.4%	647	78.2%	367	78.6%	1.215
	Others	95	7.3%	66	8.0%	29	6.2%	
Educational level								
	Illiterate/ primary education	330	25.5%	221	26.7%	109	23.3%	1.408
	Secondary education	602	46.5%	380	45.9%	222	47.5%	
	Bachelor’s or higher level	101	7.8%	66	8.0%	35	7.5%	
Employment								
	In work	568	43.9%	368	44.5%	200	42.8%	0.263
	Unemployed	336	26.0%	212	25.6%	124	26.6%	
Marital status								
	Single (unmarried, divorced)	70	5.4%	30	3.6%	40	8.6%	18.711***
	Married	348	26.9%	243	29.4%	105	22.5%	
CCMD-3 Diagnosis								
	Organic mental disorders	47	3.6%	31	3.7%	16	3.4%	16.420*
	Mental disorders due to substances	46	3.6%	37	4.5%	9	1.9%	
	Schizophrenia and other psychotic disorders	760	58.7%	449	54.3%	311	66.6%	
	Mood (affective) disorders	90	7.0%	64	7.7%	26	5.6%	
	Hysteria, Stress-related disorders, Neurosis	7	0.5%	5	0.6%	2	0.4%	
	Personality disorders, Habit and impulse disorders, Psychosexual disorders	2	0.2%	1	0.1%	1	0.2%	
	Mental retardation, and disorders of psychological development adolescence	8	0.6%	6	0.7%	2	0.4%	
	Other mental disorders and psychological health conditions	28	2.2%	13	1.6%	15	3.2%	
The type of crime involved								
	Intentional homicide	420	32.5%	244	29.5%	176	37.7%	32.410***
	Intentional assault	468	36.2%	274	33.1%	194	41.5%	
	Arson	67	5.2%	59	7.1%	8	1.7%	
	Robbery	41	3.2%	22	2.7%	19	4.1%	
	Endangering public safety	19	1.5%	13	1.6%	6	1.3%	
	Disrupting public service	16	1.2%	14	1.7%	2	0.4%	
	Intentional destruction of property	24	1.9%	18	2.2%	6	1.3%	
	Others	27	2.1%	19	2.3%	8	1.7%	
Criminal responsibility								
	No responsibility	1,293	99.9%	826	99.9%	467	100.0%	0.000
	Partial responsibility	1	0.1%	1	0.1%	0	0.0%	
Applicant								
	Person subject to mandatory treatment	60	4.6%	35	4.2%	25	5.4%	1.043
	Family members	905	69.9%	584	70.6%	321	68.7%	
	Medical treatment institutions	326	25.2%	206	24.9%	120	25.7%	
	Others	3	0.2%	2	0.2%	1	0.2%	

*p < 0.05, **p < 0.01, ***p < 0.001. Missing values and missing rates are shown in the supplementary table.

### 3.5. Information on multiple applications for relief

A total of 118 offenders with mental illness applied for relief from mandatory treatment multiple times. Table 3 presents the number of rejections and the time interval between two applications. It can be seen that 56 individuals were finally relieved from mandatory treatment. Among the 48 individuals who were rejected for first application, 39 (81.3%) had applications on both occasions made by their close relatives, 4 (8.3%) applications by medical treatment institutions, 2 (4.2%) applications by the offenders themselves, and only 3 (6.3%) applied twice and had different applicants. The interval between two applications was 11.3 ± 7.0 months.

**Table 3:** Summary statistics of multiple applications for relief by the same offender (*N* = 118).

Procedure	Total	Number of rejections	Number of offenders	Application interval (months)	
				Min	Max
Final relief of mandatory treatment	56	1	48	3	33
		2	7	2	31
		3	1	1	11
Final rejection for relief	62	2	53	2	42
		3	7	5	36
		4	2	7	35

The results showed that 62 individuals with mental disorder were still refused relief of mandatory treatment after repeatedly applying. Among the 53 individuals who were rejected twice, 33 (62.3%) applied twice through their close relatives, 14 (26.4%) had applications from medical institutions, 2 (3.8%) applied themselves, and 4 (7.5%) had applications made three times by different applicants. The application interval was 16.0 ± 10.2 months.

## 4. Discussion

This study summarized the documents of mandatory treatment from 2013 to 2021 showing there has been an improvement in the processing of mandatory treatment in China. Like many countries, China sends offenders with no criminal responsibility to receive treatment instead of punishing them by imprisonment (25, 26). This may reflect an increasing sophistication in terms of public knowledge of their rights, but also expectations for their public safety in China. In this context, the Criminal Procedure Law (implemented in 2013) can be considered as setting mandatory treatment as a special procedure in which mentally ill offenders without criminal responsibility are sent to specialist institutions for mandatory treatment, based both on the rights of patients, but also to ensure the safety of the public (27, 28).

We found that since the promulgation of the special procedure for criminal mandatory treatment, the number of cases involving mandatory treatment in courts across China increased year by year from 2013 to 2019, with the highest number of cases reaching 996 in 2019 (Figure 3). This trend of mandatory treatment cases is consistent with *Wang*’s research (29). Since the implementation of a more specific law on mandatory treatment in 2013, each stage of the mandatory treatment procedure has been made clearer, together with rules initiating forensic psychiatric assessment and initiation of mandatory treatment procedures. There was a sharp decline in the number of cases in 2020 and 2021. This may be due to the COVID-19 pandemic, shortening working hours, leading to fewer cases being posted online. Court dates for cases may also be delayed, with some mandatory treatment-related cases not being heard. It may also be that people spent more time at home during covid-19 pandemic and patients with mental disorders were monitored more effectively by their families.

Our results showed that there were significant differences in the distribution of diagnosis and criminal responsibility between the mandatory treatment group and the rejected mandatory treatment group. We found the highest proportion of diagnoses in the mandatory treatment group was “Schizophrenia and other psychotic disorders.” This is consistent with the findings of Whiting, Lichtenstein and Fazel, suggesting that patients with schizophrenia had a higher risk of violent crime (28). In the present study, all were found to have no criminal responsibility. At the same time, the results showed that there were significant differences in the distribution of the diagnosis and the type of crime involved when comparing the group who were relieved from mandatory treatment and the group who continued receiving mandatory treatment. The group who continued in mandatory treatment were more likely to be diagnosed “Schizophrenia and other psychotic disorders,” and with more crimes of intentional homicide and intentional injury. In China, the court will refer to the severity of the case and the assessment of the condition when deciding whether to reject or continue to mandatory treatment.

We found that all applications for mandatory treatment were submitted to the court by the procuratorate. In China, only the procuratorate can apply to the court for mandatory treatment, and the people’s court is the only body that can initiate the mandatory treatment. In the USA, the initiation right is that of the parties themselves and their legal representatives (30). However, we found that the court combined multiple information to decide whether to order mandatory treatment or not. The information included the respondent’s mental history, treatment history, whether there had been violent injuries, the forensic psychiatric assessment opinion, whether his/her close relatives had the capacity to supervise, the offenders’ current condition, treatment, and needs for control.

The results of our study were consistent with China’s legal provisions, reflecting the scope of application for mandatory treatment: that is, mentally ill offenders who commit acts of violence and are identified by legal procedures as not criminally responsible according to law, but who may still endanger public safety and the personal safety of citizens (31). In many countries, mandatory treatment procedures apply to a wider range of subjects. For example, the German Criminal Code stipulates that the application objects of mandatory treatment not only include mentally ill offenders without criminal responsibility, but also covers those with partial criminal responsibility. The application objects of the mandatory treatment procedure in Britain are not only those who are acquitted after the successful defense of mental illness, but includes those who are considered mentally unfit to stand trial, enter a plea of guilty or not guilty, and need medical treatment, as well as those who suffer from mental illness during their imprisonment (26).

Our results showed that a proportion of the parties or relatives were dissatisfied with the trial decision of mandatory treatment, so they applied to the higher court for reconsideration of the mandatory treatment decision. The reconsideration decisions we collected show that the reasons for applying for reconsideration were mainly the improvement of the offender’s condition, the guardian having supervisory capacity, and questioning the validity of the forensic psychiatric assessment or other evidence considered at the original judgment. This differs from the appeal process of the USA (32), Chinese law regards the process of reconsideration as a mechanism which lifts the legal burden on the patient after the initiation of mandatory treatment procedures. It is more in line with China’s national conditions and also conducive to the protection of the legitimate rights and interests of the offenders receiving mandatory treatment (29). On the one hand, the outcome of a formal reconsideration reflects the fairness and effectiveness of the court judgment, on the other hand, it also protects the human rights of the offender subjected to mandatory treatment.

Our results showed that only 64% of the cases applying for relief from mandatory treatment were granted relief. In China, after being subjected to mandatory treatment, there are corresponding procedures for applying for relief of mandatory treatment. It is ultimately up to the court to decide whether to relieve, as is the case in the United Kingdom. However, some states in the USA have also given the right of relieving mandatory treatment to hospitals (33). Article 288 of the Criminal Procedure Law (implemented in 2013) stipulates that the institution responsible for mandatory treatment institution, the parties, and their close relatives are the applicants for relief of mandatory treatment. Most of the applications for relief were submitted by family members, whether patients were relieved of mandatory treatment or needed to continue. The research results showed that close relatives were more likely to apply for relief of mandatory treatment (34). In 2013, virtually no one applied for relief, and the number of relief applications then increased year by year (Figure 3). The increase in number of applications for relief from mandatory treatment is considered to be related to the recovery of the illness after a period of mandatory treatment, as well as the willingness of family members to accept and care for patients.

We found wide variation across documents in the duration of mandatory treatment, from 1 month to 96 months. Chinese law has no clear stipulations on the specific time allowed for mandatory treatment (26). As long as the person subjected to mandatory treatment is no longer dangerous after treatment, mandatory treatment can be relieved. The main basis of a decision is the diagnostic evaluation report issued by the responsible treatment institution. Other evaluation evidences include the transcripts of the investigation and interview report from the medical treatment institution, the statements of the applicant and the respondent, the letter of commitment of relatives’ guardianship, and the forensic psychiatric assessment opinion again if necessary. The standards of courts in different provinces and cities are basically the same (34). Germany, which also has a civil law system, can institutionalize patients with mental disorder without criminal responsibility for an indefinite period (29), Switzerland determines the type and maximum period of mandatory treatment by comprehensively considering harmful acts and infringement of legal interests (17). Some states in the United States, such as California, stipulate in their criminal code that the period of mandatory treatment for a mentally ill offender who is not criminally responsible must be within the maximum prison term for the crime committed. If the period of mandatory treatment needs to be extended, a hearing must be held (35).

## 5. Limitation

This study has certain limitations. First, due to privacy protection, it is impossible to obtain all the information on offenders who receive mandatory treatment from available documents on the China Judgments Documents Online. There are missing values for some items. However, we conducted a large sample analysis to reduce bias. Second, we can only make statistical analysis of the published documents, which cannot cover all mandatory treatment cases. Third, the information in the judgment document is static and relatively simple, so we can only summarize it based on the existing information.

## 6. Conclusion

This study describes mandatory treatment procedures for mentally ill offenders with no criminal responsibility in court in China. The conclusions from different types of documents show that there are different approaches to the disposition of mentally ill offenders. Both mandatory treatment and relief from mandatory treatment should be handled in strict accordance with the law and special procedures. This is conducive to protection of social security and protection of the rights of mentally ill offenders.

## Data availability statement

Publicly available datasets were analyzed in this study. This data can be found at: https://wenshu.court.gov.cn/.

## Author contributions

Y-FQ and X-TY extracted the data, reviewed the documents, analyzed the data, and wrote the manuscript. Z-YW and RY reviewed and checked the content and format of the manuscript. JC and X-CC reviewed the manuscript and revised the article for important intellectual content. J-MH and X-CC conceived and designed the study. All authors contributed to the article and approved the submitted version.

## Funding

This study was supported by the China Postdoctoral Science Foundation (Grant no. 2020TQ0219).

## Conflict of interest

The authors declare that the research was conducted in the absence of any commercial or financial relationships that could be construed as a potential conflict of interest.

## Publisher’s note

All claims expressed in this article are solely those of the authors and do not necessarily represent those of their affiliated organizations, or those of the publisher, the editors and the reviewers. Any product that may be evaluated in this article, or claim that may be made by its manufacturer, is not guaranteed or endorsed by the publisher.

## Footnotes

^1^http://wenshu.court.gov.cn/
