# Supplementary material for: Status of mandatory treatment of mentally ill offenders without criminal responsibility in China: Information from 5,262 mandatory treatment judgments
Source: Front Psychiatry. 2023 Apr 3;14:1129954. doi: 10.3389/fpsyt.2023.1129954 (PMC10106559; doi:10.3389/fpsyt.2023.1129954)
Supplement: Supplementary file 1 [file Table_1.docx]

Supplementary table The missing value and missing rate of basic information of mentally ill offenders in various documents, N(%)

|  | Applied for mandatory treatment（total） | Decision mandatory treatment | Decision to reject mandatory treatment | Application to relieve mandatory treatment（total） | Decision to relieve mandatory treatment | Decision to reject relief of mandatory treatment | Decision documents for reconsideration mandatory treatment decision |
| --- | --- | --- | --- | --- | --- | --- | --- |
| **Sex** | 251（6.5%） | 244（6.5%） | 7（6.5%） | 65（5.0%） | 48（5.8%） | 17（3.6%） | - |
| **Age** | 588（15.3%） | 569（15.2%） | 19（17.8%） | 179（13.8%） | 118（14.3%） | 61（13.1%） | - |
| **Nationality** | 596（15.5%） | 581（15.5%） | 15（14.0%） | 185（14.3%） | 114（13.8%） | 71（15.2%） | - |
| **Educational level** | 752（19.5%） | 734（19.6%） | 18（16.8%） | 261（20.2%） | 160（19.3%） | 101（21.6%） | - |
| **Employment** | 926（24.0%） | 894（23.9%） | 32（29.9%） | 390（30.1%） | 247（29.9%） | 143（30.6%） | - |
| **Marital Status** | 2482（64.4%） | 2420（64.6%） | 62（57.9%） | 876（67.7%） | 554（67.0%） | 322（69.0%） | - |
| **CCMD-3 Diagnosis** | 235（6.1%） | 232（6.2%） | 3（2.8%） | 306（23.6%） | 221（26.7%） | 85（18.2%） | 7（6.1%） |
| **The type of crime involved** | 7（0.2%） | 5（0.1%） | 2（1.9%） | 212（16.4%） | 164（19.8%） | 48（10.3%） | 4（3.5%） |
| **Duration of treatment** | - | - | - | 8（0.6%） | 4（0.5%） | 4（0.9%） | - |
